# Genome-wide motif predictions of BCARR-box in the amino-acid repressed genes of *Lactobacillus helveticus* CM4

**DOI:** 10.1186/s12866-017-1125-0

**Published:** 2017-12-02

**Authors:** Naoyuki Yamamoto, Taketo Wakai

**Affiliations:** 1 School of Life Science and Technology, Tokyo Institute of Technology, 4259-J3-8, Nagatsuta-cho, Midori-ku, Yokohama, Kanagawa 226-8501 Japan; 2 Research and Development Center, Asahi Group Holdings Ltd., 11-10, 5-chome, Fuchinobe, Chuo-ku, Sagamihara-shi, Kanagawa 252-0206 Japan; 3 Core Technology laboratories, Asahi Group Holdings Ltd., 11-10, 5-chome, Fuchinobe, Chuo-ku, Sagamihara-shi, Kanagawa 252-0206 Japan

**Keywords:** BCARR, *Lactobacillus helveticus*, Genome-wide search, BCARR-box, MEME analysis, Palindromic structure

## Abstract

**Background:**

A BCARR (branched-chain amino acid responsive repressor) identified in proteolytic gene expressions in *Lactobacillus helveticus* is considered to negatively control transcriptions by binding to operator sites at the promoter regions in the presence of BCAAs. However, the distributions and regulatory potential of the BCARR in all genes repressed by BCAAs in CM4 remains unclear.

**Results:**

A genome-wide search for the BCARR-box was conducted to clarify the contribution of BCARR in the regulation of amino acid metabolism in *L. helveticus* CM4. Among all 2174 genes of CM4, 390 genes repressed by amino acids were selected for the search of the BCARR-box. The annotated 33 genes among the 67 predicted BCARR-boxes were mainly linked to amino acid metabolism. The BCARR-boxes were mainly located adjacent to the −35 sequence of the promoter; however, the repressive effects in different locations were similar. Notably, the consensus BCARR-box motif, 5′-A1A2A3A4A5W6N7N8N9W10T11T12W13T14T15–3′, observed in highly repressed genes, revealed more frequent A-T base pairing and a lower free energy than that in lowly repressed genes. A MEME analysis also supported the lower frequency of T at positions 12, 14, 13 and 15 in the BCARR-box sequence of the lowly repressed gene group. These results reveal that genes with a more stable palindromic structure might be preferable targets for BCARR binding and result in higher repressions in the target gene expressions.

**Conclusions:**

Our genome-wide search revealed the involvement of the proteolytic system, transporter system and some transcriptional regulator systems in BCARR-box regulation in *L. helveticus* CM4.

**Electronic supplementary material:**

The online version of this article (10.1186/s12866-017-1125-0) contains supplementary material, which is available to authorized users.

## Background

The proteolytic system of lactic acid bacteria is crucial for cell growth in milk and important for the acceleration of ripening in cheesemaking and rapid yogurt manufacturing. The proteolytic system is activated at the beginning of fermentation to release peptides and amino acids for cell growth because of limited nitrogen sources in milk, but is negatively controlled by accumulated amino acids and peptides at the late phase of cell growth [1, 2].

Generally, lactobacilli have stronger proteolytic activities and can release higher amounts of peptides and amino acids in fermented milk compared with lactococci [3]. Among *Lactobacillus* species, *Lactobacillus helveticus* has the highest proteolytic activity and can release antihypertensive peptides from casein during the milk fermentation process [3–6]. *L. helveticus* CM4 with the highest proteolytic activity can release the highest amount of these peptides [7, 8]. However, the production of the antihypertensive peptides by *L. helveticus* CM4 was repressed by amino acids that accumulated in fermented milk because of the down-regulation of genes, such as *pepO2*, *pepCE* and *pepE*, that most likely encode enzymes involved in the processing of active peptides [7, 9]. A novel type of regulator protein, a cystathionine β-synthase (CBS) domain protein involved in the regulation of the proteolytic system, was successfully identified in a previous study [10]. The CBS domain protein binds to a specific DNA sequence present at the promoter regions of the repressed proteolytic genes in response to intracellular BCAAs [10]. From a comparative sequence analysis of the promoter regions of the proteolytic genes, a gel shift assay and a footprinting analysis, a palindromic AT-rich motif, 5′-AAAAANNCTWTTATT-3′, was predicted as the consensus DNA motif for the branched chain amino acid responsive repressor (BCARR) protein binding box (BCARR-box). Therefore, the consensus DNA motif is thought to exist in many genes repressed by amino acids including those of the proteolytic enzymes of CM4 [9], but the contributions of BCARR via binding to the BCARR-box in the repressed genes of CM4 are unclear. In *Lactococcus lactis* and *Bacillus subtilis* most of the proteolytic genes are regulated by the CodY protein in response to branched chain amino acids (BCAAs) [1, 11–14]. CodY is activated by binding to accumulated BCAAs in the medium, which increases the affinity to its operator site, the CodY protein binding box (CodY-box) [11–14]. However, no CodY and no regulatory system of the proteolytic enzyme have been reported.

Genome-wide search is a powerful tool to understand the contribution of the regulatory system in specific gene expressions in response to some metabolites [15–19]. In the present study, we searched the BCARR-box previously predicted from six kinds of proteolytic genes which were down-regulated in response to amino acids in CM4 [10]. Then, we characterized the structural features of the BCARR-box, palindromic pair, free energy and location from the promoter, to determine the BCARR-driven repressed effect. We also investigated the impact of the BCARR system on amino acid metabolism, which plays a crucial role in cell growth in milk and other metabolism throughout the selection of the down-regulated genes by amino acids.

## Results

### Distribution of the repressed genes in the whole CM4 genome

**Fig. 1:**
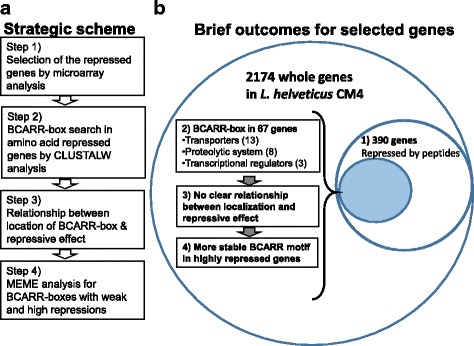
Strategic workflow of the BCARR motif search and analysis (**a**) and brief outcomes (**b**). **a** Four-step analytical process from a motif search in the repressed genes (Step 1) to a motif analysis for repressive effects (Step 4). **b** Brief outcomes of each workflow step are summarized

**Fig. 2:**
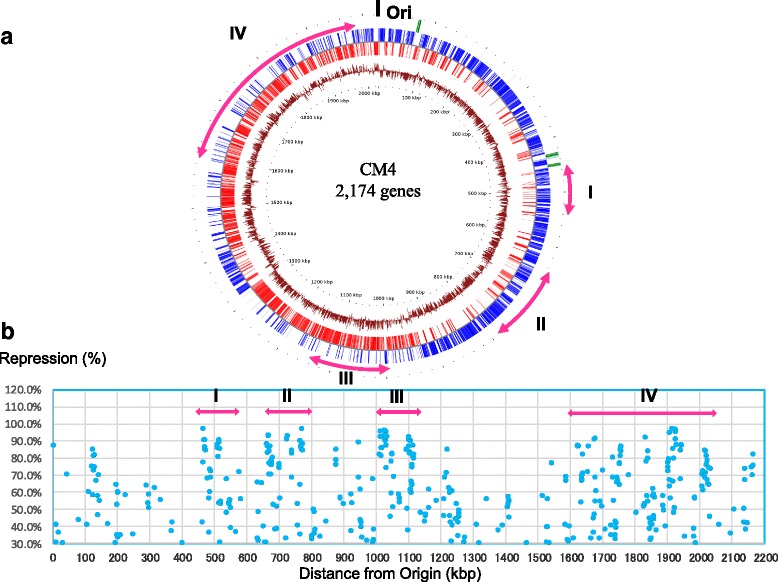
Genome-wide distribution of the repressed genes of *Lactobacillus helveticus* CM4 in response to amino acids on the genome map (**a**) and locations from the origin with the repressive effects (**b**). **a** Location of the repressed genes by amino acids on a previously reported physical map of *Lactobacillus helveticus* CM4 chromosomal DNA [20]. Regions of highly repressed genes (I to IV) are shown on the physical map. Maps on the outside: Positions of tRNA (pale blue), rRNA operons (green), ORFs on the positive strand (blue), ORFs on the negative strand (red) and GC-skew plot (brown). Distances (kbp) from the origin are shown on a dotted line on the inside. **b** Locations of the repressed genes from the origin and the repressive effects by amino acids in CM4. Regions of highly repressed genes (I to IV) are also shown in these maps. The raw data is provided in Additional file 1: Table S1

Strategic steps to determine the contribution of BCARR on the regulation of specific gene expressions by amino acids in *L. helveticus* CM4 and the brief outcomes obtained at each step are illustrated in Fig. 1. In the genome-wide transcriptomic analysis, 390 genes of *L. helveticus* CM4 repressed over 30% at 0.5 h after the addition of peptides in fermented milk were observed (Additional file 1: Table S1). Various kinds of genes, such as protease, transporter, nuclease and regulatory protein genes, were repressed (detailed in Table S1). Among the 390 repressed genes, 185 genes (47.4%) encoded putative and unknown proteins. To visualize the genome-wide distributions of repressive gene expressions in *L. helveticus* CM4, the locations of the repressed genes from the origin of the CM4 genome and the repressive effects by peptides are illustrated in Fig. 2. Various genes with different repressive effects and different locations were positioned in the whole genome (Fig. 2b). Notably, highly repressed genes were located mainly in four large regions (from I to IV in Fig. 2b) and in the previously reported whole genome of *L. helveticus* CM4 (Fig. 2a) [20].

### Prediction of BCARR-box in repressed genes

As the preliminary study for whole genes, the genome-wide survey focused on the 390 genes down-regulated by amino acids. The homologue for an AT-rich palindromic motif, 5′- AAAAANNCTWTTATT -3′, predicted as the consensus DNA motif from 6 proteolytic genes in a previous study [10], was surveyed in promoter regions at −300 to 250 bp from the −35 sequence of the promoter region in 390 repressed genes by multiple sequence alignment with a CLUSTALW analysis (http://www.genome.jp/tools-bin/clustalw). In all, 67 kinds of predicted BCAA-boxes were found in the repressed genes at the promoter regions (Table 1). Corresponding genes, the observed BCARR-box sequence, distances from the −35 sequence of the promoter and the repressive effects by peptides are listed in Table 1. Strands with an observed BCARR-box and the Waterman-Eggert score analyzed by LALIGN analysis (http://www.ch.embnet.org/software/LALIGN_form.html) are also listed in Table 1. There were no significant differences in the repressive effects of BCARR-boxes located between plus and minus strands. All six proteolytic genes had BCARR-box sequences, but only *pepO2*, *pepD*, *pepC2* and *dppD* genes with repressive effects of 93.0%, 89.0%, 68.0% and 34.0%, respectively, are listed in Table 1; *pepV* and *pepO* genes showed lower repressive effects (27% and 25%, respectively).

**Table 1:** BCARR-boxes observed in promoter regions of specific genes and repressed gene expression in presence of peptides

No	ORF No	Repression (%)	Strand	^a^ Score	Location (bp)	Gene	Function	BCARR-box sequence
1	464	97.0	–	35	−11	*dapF*	Diaminopimelate epimerase	AAAATCACTTTTTTA
2	1025	97.0	+	46	254	*paaD*	Predicted metal-sulfur biosynthetic enzyme	AAAAATGATATTATC
3	1029	95.0	+	56	7	*hisM*	ABC-type amino acid transportor	AATAAGACTATTATT
4	724	93.0	+	60	87	*pepO2*	Neutral endopeptidase	AAAAAATGCTTTTAT
5	726	93.0	+	37	−120	*potE*	Amino acid transporters	AAAAAATCATGTTTT
6	1032	93.0	+	37	−229	*ddpA*	ABC-type dipeptide transport system	AAAATTCTAAAATAT
7	765	91.0	–	33	116	*himM*	ABC-type amino acid transporter	AAAAGTATTGTCTTT
8	769	91.0	+	45	−116	*putative*	unknown	AAAAAATCTATTTTT
9	1099	91.0	–	37	−19	*med*	Uncharacterized ABC-type transport system	GAAAATAATGTTCTT
10	1104	91.0	+	42	150	*putative*	unknown	AAAAAAGCCATTCTT
11	513	90.0	+	42	24	*nlpA*	ABC-type metal ion transport	AGAAATACAATTATT
12	661	89.0	+	37	−16	*pepD*	Di- and tripeptidases	AATAGACTTTTTTAT
13	1113	87.0	+	42	41	*purK*	Phosphoribosylaminoimidazole carboxylase	AAAATACCTTGTATT
14	1621	87.0	–	33	92		Uncharacterized conserved protein	AATACAAGATATTGT
15	670	86.0	–	33	−11	*putative*	unknown	AGAAATAGATTTTTT
16	738	85.0	+	35	42	*putative*	unknown	AAAGTAAGCGTTTTA
17	1219	85.0	+	46	35	*prtH, prtP*	Protease P&H	AAAAAATTAAATGTA
18	123	85.0	–	38	−15	*putative*	unknown	ACAAAAATTATTCTT
19	1102	84.0	+	48	−254	*putative*	unknown	TAAAAAAATATTATT
20	737	83.0	–	36	74	*hpt*	Hypoxanthine-guanine phosphoribosyltransferase	TGAAAAAGTATTATT
21	1543	77.0	–	34	−104	*guaA*	GMP synthase	AACAAGAACTTTTTG
22	129	75.0	+	45	−14	*putative*	unknown	AAAAATCCGTTTTTT
23	1030	74.0	+	56	−47	*putative*	unknown	AATAATAGTCTTATT
24	576	72.0	–	36	−135	*potE*	Amino acid transporters	GAAGAATACTCTTTG
25	1216	72.0	–	55	−29	*putative*	unknown	AATAAAAGGTTTTTA
26	1634	70.0	+	47	−218		ABC-type transportor	AAAAAAACTTTCCTA
27	1586	69.0	+	35	−14	*guaB*	IMP dehydrogenase	AACAAGTCCTTTTTT
28	1212	69.0	+	51	64	*pepN2*	Aminopeptidase N2	AGAACAACACTTTTA
29	1541	68.0	+	33	−90	*putative*	unknown	AACAAAACGATCATT
30	536	68.0	+	46	−67	*pepC2*	Aminopeptidase C	AAAATGCCAATTATT
31	1585	67.0	+	38	−3	*pepE*	Aminopeptidase C	GAACCCGCTTTTATT
32	632	66.0	–	46	2	*putative*	unknown	AAAAAGTCCAATCTT
33	646	65.0	+	40	−89	*putative*	unknown	AAACGGACAACCTTT
34	1162	62.0	–	42	−135	*baeS*	Signal transduction histidine kinase	AATTAAAGGTTTTAT
35	950	62.0	+	44	10	*ddpA*	ABC-type dipeptide transportor	AAAACATGGTATTAT
36	668	62.0	–	37	−21	*putative*	unknown	AAAAAGCAGCTTAGT
37	691	59.0	+	31	−177	*lytT*	Response regulator	AAAATCTCGCTTTTT
38	665	59.0	–	33	−90	*putative*	unknown	AAATATGATATTTTT
39	296	59.0	+	33	−46	*uup*	ATPase components	AAAAAGTTTTAATTA
40	1070	59.0	–	46	60	*putative*	unknown	AATAACACTGTTTTT
41	1514	58.0	–	38	−29		GAF domain-containing protein	AATCAAACTTTTTTC
42	701	57.0	–	37	−30		Uncharacterized conserved protein	GAGAAAAGGTTTGTT
43	1529	56.0	–	40	−89		FOG: CBS domain-containing protein	AAGAGATGCTTTTTT
44	1340	56.0	–	46	39	*pepQ*	Xaa-Pro aminopeptidase	AAAAAGAGGCTATTT
45	579	56.0	+	42	125	*uvrC*	Nuclease subunit	AAAAACCGGGCTGTT
46	518	55.0	+	46	−29	*putative*	unknown	AAAAAAACAATTATA
47	755	53.0	–	39	99	*folB*	Dihydroneopterin aldolase	AATACGAGGAGTTTT
48	1051	52.0	+	40	49	*putative*	unknown	AATCAATGTATTATT
49	1406	51.0	–	49	−51	*fatA*	Acyl-ACP thioesterase	AATATAAGTCCTTTT
50	806	50.0	+	57	64	*putative*	unknown	AAAAGAAGCTTGTTT
51	807	49.0	+	40	27	*putative*	unknown	AACAATAGAATTATA
52	537	48.0	+	38	−1	*citT*	Di- and tricarboxylate transporters	AAAAATAACTTTATT
53	1208	47.0	–	34	64	*hflC*	Membrane protease subunits	AACAACAGGACTTTA
54	1151	46.0	+	43	−122	*putative*	unknown	AAAAAATTTTTTTGT
55	1254	46.0	+	49	95	*putative*	unknown	AAAATAACTATTAAT
56	669	44.0	–	34	−222	*putative*	unknown	ATGAAATCGCTTATT
57	1530	40.0	+	42	149	*putative*	unknown	AAAAAAGCTTTGCTT
58	921	38.0	+	44	−12	*putative*	unknown	AAAACATCTTTTTTG
59	763	35.0	+	35	55	*putative*	unknown	AAAAATGCTTGACTT
60	201	34.0	+	36	109	*deoR*	Transcriptional regulator	AAAAATACATTGTTA
61	825	34.0	–	47	−8		Uncharacterized protein	ATTAATAGGTTTTCT
62	954	34.0	+	49	59	*dppD*	ABC-type dipeptide transportor	AAAAAAGCTATAAAT
63	1593	32.0	+	46	124	*lytR*	Transcriptional regulator	AAAAAACGTCATATT
64	635	32.0	+	51	−19	*mdlB. cydC, sunT*	ATPase and permease components	AAAAACACTTTAATT
65	1590	32.0	+	40	−29	*putative*	unknown	AAAACAGTTTTCTTA
66	655	31.0	+	41	167	*putative*	unknown	AAAAAACGTTTTAGC
67	1535	30.0	–	36	114	*putative*	unknown	AAAAGTAGTTTTCAT

^a^ Waterman-Eggert score by LALIGN analysis

### Predicted genes with a BCARR-box

**Fig. 3:**
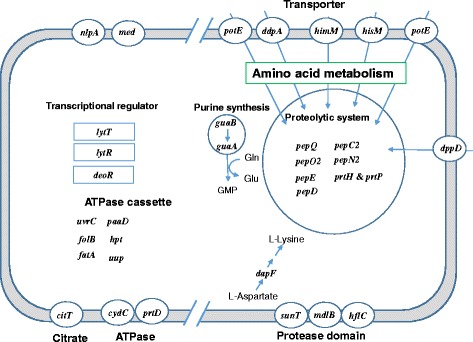
Down-regulated genes with BCARR-box sequences at the promoter regions in *Lactobacillus helveticus* CM4. Genes suggested to be involved in amino acid metabolism, the proteolytic system including related transporters and membrane proteases are shown at the right with linked arrows

To understand the role of the BCARR in the regulatory system by amino acids, annotated genes with observed BCARR-boxes (Table 1) are summarized in Fig. 3. Among the 67 predicted BCARR-boxes in the promoter regions of the 390 repressed genes, 34 genes were uncharacterized or non-annotated genes listed in Table 1. Half of the annotated 33 genes were linked to amino acid metabolism, such as transporters, proteolytic system and purine synthesis. Nine genes (*potE, ddpA, himM, hisM, potE, dppD*, *sunT*, *mdlB* and *hflC*) were transporter genes. Nine genes (*pepO, pepO2, pepE, pepV, pepQ, pepC2, pepN2, prtH&P* and *pepD*) (reviewed in ref. [7]) were proteolytic enzymes. *LytT*, *lytR* and *deoR* were regulator genes (see Fig. 3). The g*uaA* and *guaB* genes for purine synthesis [21] may have a link to amino acid metabolism.

Changes of transcriptional regulators by amino acids, such as *lytL*, *lytR* and *deoR*, are of interest because these regulators may impact many gene expressions indirectly through BCARR action. The gene products of transcriptional regulators *lytL* and *lytR* have been suggested to influence *alaD* gene expression [22]. *DeoR*, which is widely present in bacteria and acts as a repressor in sugar metabolism [23], may have an indirect effect on sugar metabolism. Various transporter genes have BCARR-motifs at the promoter regions. The *sunT* gene product has been suggested to function as an antibiotic transporter with a protease domain [24, 25]. The *mdlB* gene product also has a protease domain and is likely involved in multidrug transport and bacteriocin export [26]. The *hflC* product also has a protease domain and is involved in protein secretion [27]. The *cydC* gene product co-expressed with the *cydD* gene in *E. coli* showed ATPase-like activity [28]. PrtD is one of the ATP binding cassette components with low ATPase activity involved in the protease secretion system [29]. CitT is a component of the two-component system that plays a crucial role in citrate utilization [30].

### Localization and the repressive effects of the BCARR-box

**Fig. 4:**
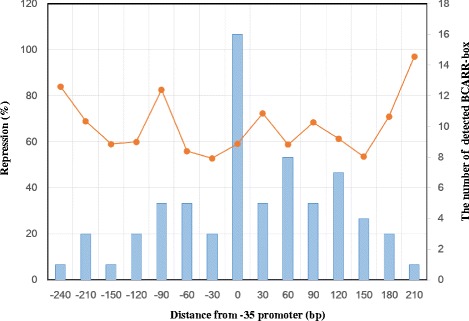
The number of predicted BCARR-boxes and the average repressive activities with different locations. Data collected from predicted motifs located in each 30 bp long sequence were used to calculate the average repressive effects (indicated by a line). The number of detected BCARR-boxes is shown by a column. The locations indicated are the distances (bp) from the −35 bp sequence of the promoter: −240 (from −269 to −240 bp), −210 (from −239 to −210 bp), −150 (from −179 to −150 bp), −120 (from −149 to −120 bp), −90 (from −119 to −90 bp), −60 (from −89 to −60 bp), −30 (from −59 to −30 bp), 0 (from −29 to 0 bp), 30 (from 1 to 30 bp), from 60 (from 31 to 60 bp), 90 (61 to 90 bp), 120 (from 91 to 120 bp), 150 (from 121 to 150 bp), 180 (from 151 to 180 bp) and 210 (from 181 to 210 bp)

To understand how the location of the BCARR-box at the promoter regions could interfere with RNA polymerase–promoter binding and the transcriptional activity of the underlying gene, the distance of the predicted boxes from the origin of the CM4 genome and the repressive effects are summarized in Fig. 4. Most of the BCARR-boxes were present between −120 to +150 bp from the −35 sequence of the promoter, and the boxes were most frequently observed at 0 bp (−30 to 0 bp). Unexpectedly, the average repressive effects of each box with different locations at the promoter regions were almost similar (Fig. 4). This result indicates that a wide promoter region, not the more frequent BCARR-box adjacent to the −35 sequence, might be sufficient to express the repressive effects on the transcription.

### Comparison of highly and lowly repressive box sequences

**Fig. 5:**
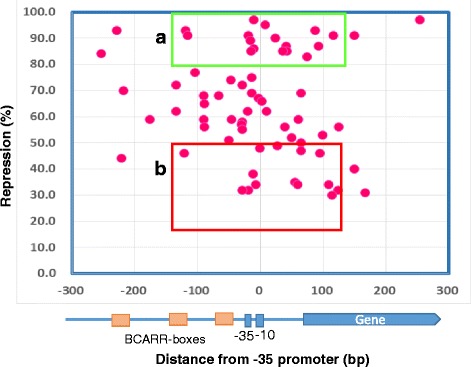
Location of the predicted BCARR-box in the repressed genes and the repressive effects. The distances from the **−**35 bp sequence of the promoter (bp) and the repressive effects listed in Table 1 are illustrated in this Fig.. **a**: A highly repressed motif located −120 to +150 bp from the −35 bp sequence of the promoter and with repression over 80% (green box). **b**: A lowly repressed motif located −120 to +150 bp from the −35 bp sequence of the promoter and with repression from 50% to 30% (red box)

No clear differences in average repressive effects that were dependent on the location of BCARR-boxes in the promoter regions were observed if the regions were limited from −300 to +250 bp from the −35 sequence (Fig. 4). Therefore, each location of the BCARR-box from the −35 sequence of the promoter and the repressive effects are illustrated in Fig. 5. BCARR-boxes were most frequently present at −120 to +150 bp from the −35 bp sequence of the promoter. Thus, BCARR-boxes from −120 to +150 bp with high and lowly repressions were selected for structural feature analysis. As listed in Table 2, the repressive effect for Group A shown by green box with repression over 80% was 88.9 ± 3.9%, and that of the low repressive Group B with repression less than 50% was 36.3 ± 3.8%. The repressive effect for Group A was significantly higher than that for Group B (*P* < 0.001).

**Table 2:** BCARR-boxes observed in high and low repressive genes in response to peptides

NO	Repression (%)	ORF No	Gene	BCARR-box sequence	Palindromic pair	ΔG (kcal/mol)
<Highly repressed genes>						
1	91.0	1099	*med*	GAAAATAATGTTCTT	4	1.15
2	91.0	1104	putative	AAAAAAGCCATTCTT	5	−4.2
3	89.0	661	*pepD*	AAAAATACTTTAATT	4	0.61
4	91.0	765	*himM*	AAAAGTATTGTCTTT	3	−0.64
5	87.0	1621	putative	AATACAAGATATTGT	3	−3.6
6	93.0	724	*pepO2*	AAAAAATGCTTTTAT	5	−0.74
7	84.0	1102	putative	TAAAAAAATATTATT	4	0.94
8	83.0	737	*hpt*	TGAAAAAGTATTATT	3	0.86
9	93.0	726	*potE*	AAAAAATCATGTTTT	5	−0.44
10	91.0	769	putative	AAAAAATCTATTTTT	5	−2.45
11	93.0	1032	*ddpA*	AAAATTCTAAAATAT	4	0.18
12	85.0	738	putative	AAAGTAAGCGTTTTA	3	1.66
13	87.0	1113	*purK*	AAAATACCTTGTATT	4	−1.05
14	85.0	1219	*prtH, prtP*	AAATTAAATGTATTT	5	1.71
15	90.0	513	*nlpA*	AGAAATACAATTATT	4	0.58
16	85.0	123	putative	ACAAAAATTATTCTT	5	0.95
17	86.0	670	putative	AGAAATAGATTTTTT	4	0.04
18	97.0	464	*dapF*	AAAATCACTTTTTTA	4	0.17
AV	88.9				4.1	−0.24
SD	3.9				0.8	1.68
<Lowly repressed genes>						
1	49.0	807	putative	AACAATAGAATTATA	4	0.67
2	48.0	537	*citT*	AAAAATAACTTTATT	4	0.26
3	41.0	1582	putative	TAATAAACTGTACTT	4	1.77
4	38.0	921	putative	AAAACATCTTTTTTG	4	0.25
5	35.0	763	putative	AAAAATGCTTGACTT	2	
6	34.0	825	putative	ATTAATAGGTTTTCT	3	0.95
7	32.0	635	*mdlB, cydC, sunT*	AAAAACACTTTAATT	4	1.26
8	32.0	1590	putative	AAAACAGTTTTCTTA	3	
9	27.0	745	*pepV*	AAAAACAAATTTATT	4	−0.04
10	25.0	924	*pepO*	AAAAAACCTGTTAAC	2	0.86
11	24.0	2167	*prtD*	AAAAAATCCTTTCTT	5	0.75
12	34.0	954	*dppD*	AAAAAAGCTATAAAT	2	
13	47.0	1208	*hflC*	AACAACAGGACTTTA	2	2.14
14	46.0	1254	putative	AAAATAACTATTAAT	3	0.08
15	34.0	201	*deoR*	AAAAATACATTGTTA	3	0.76
16	32.0	1593	*lytR*	AAAAAACGTCATATT	3	2.75
17	30.0	1535	putative	AAAAGTAGTTTTCAT	3	
18	46.0	1151	putative	AAAAAATTTTTTTGT	5	−0.56
AV	36.3				3.3	0.85
SD	8.1				1.0	0.90

To determine the reason for the different repressive effects in the two groups, the number of base pairs in the palindromic sequence and the free energy for each BCARR-box were analyzed (Table 2). The average number of palindromic pairs was significantly higher in Group A (4.1 ± 0.8) than in Group B (3.3 ± 1.0) (*P* < 0.05) because of fewer Ts at positions 12, 13, 14 and 15 in Group B than in Group A. The free energy represented as the ΔG value for a BCARR-box was evaluated by M-fold analysis (http://unafold.rna.albany.edu/?q=mfold/DNA-Folding-Form) and compared among the two groups. As for the ΔG analysis, all BCARR-box sequences in Group A were available in the M-fold tool, but approximately half of the sequences in Group B were not (Table 2) As expected from the number of base pairs shown in Table 2, the ΔG values, which reflect the stability of the palindromic pair, were significantly lower in Group A (−0.25 ± 1.67) than in Group B (0.85 ± 0.90) (*P* < 0.05). The above findings revealed that the predicted palindromic sequence might be more stable for Group A than for Group B.

**Fig. 6:**
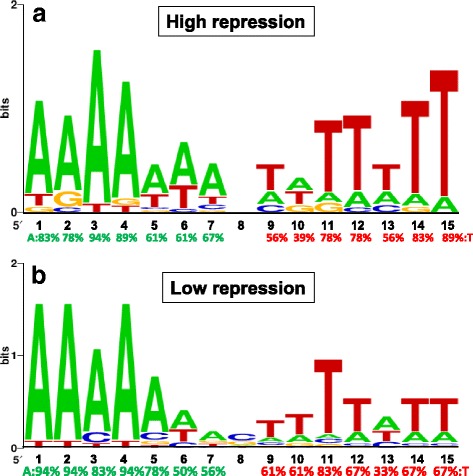
Search for consensus motifs of BCARR-boxes for highly (**a**) and lowly (**b**) repressive groups. The weight matrix shows the frequency of A, C, T or G nucleotides (as indicated in the legend) at each position of the motif. The frequencies of A and T (%) are shown in green and red, respectively, below the matrix. A graphical representation of the identified motif was obtained at the Weblogo website (http://weblogo.berkeley.edu/logo.cgi)

For further consideration of the different repressive effects between the 2 groups, the predicted motifs of the BCARR-box from 18 sequences of Group A and 18 sequences of Group B were compared by MEME analysis. As shown in Fig. 6, the structural features of the motifs for Group A were relatively conserved AT-rich palindromic sequences, but Group B contained slightly fewer Ts than Group A at positions 12 (67%), 13 (33%), 14 (67%) and 15 (67%). This result suggests that fewer Ts at positions 12, 13, 14 and 15 in the BCARR-box of Group B might make a less stable palindromic structure than that of Group A. A more stable palindromic structure of the BCARR-box in Group A genes than in Group B might have the advantage of conferring a higher affinity to the BCARR protein and, thus, a higher repressive effect. Considering the above results, the repressive effects of amino acids through the BCARR system might be more dependent on an AT-rich stable palindromic structure than on the location within the promoter region.

### CodY-box in BCARR-box regions

**Fig. 7:**
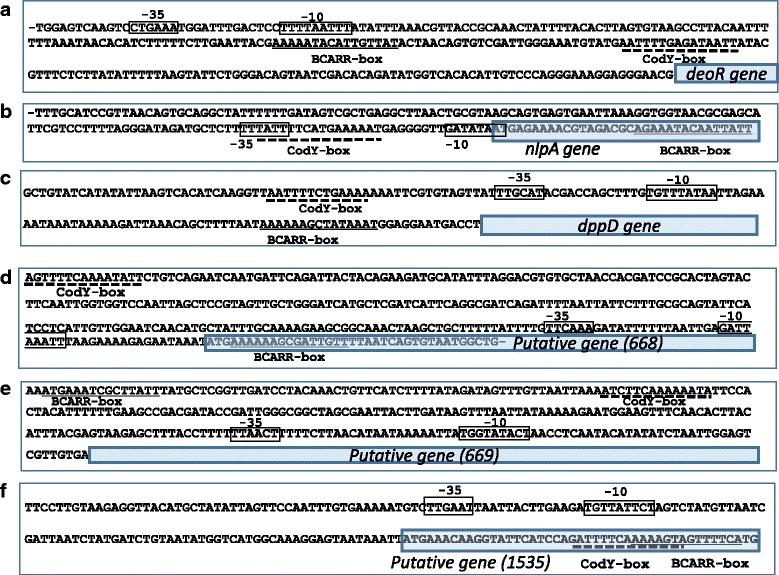
Both a CodY-box and a BCARR-box in the promoter regions of 6 genes. *deoR* (**a**), nlpA (**b**) and dppD (**c**) genes and three putative genes with ORF NOs 668 (**d**), 669 (**e**) and 1535 (**f**) are shown. The CodY-box and BCARR-box in the upstream regions of six genes are underlined by dotted and solid lines, respectively. The -35 and -10 bp sequences of the promoter sites are shown in boxes

In lactococci, CodY plays a crucial role in exerting negative regulation on proteolytic gene expressions by binding to the CodY-box in the presence of amino acids. However, no c*odY* gene has been observed in the *Lactobacillus* genome including that of CM4, and there is no information in the literature about a CodY-box sequence, 5′-AATTTTCWGAAAATT-3′, in lactobacilli. On the other hand, *B. subtilis* has both CodY and BCARR genes, suggesting a regulatory system response to BCAAs [14]. So, to investigate the evolutional selection of the regulatory system in lactobacilli, a CodY-box sequence was searched at the promoter regions of 67 genes with BCARR-box sequences in CM4 (Table 1). A CLUSTALW analysis showed that most of the upstream DNA sequences had no CodY-box sequence, however, both the BCARR-box and CodY-box sequences were observed in *deoR*, *nlpA* and *dppD* genes and three putative genes at the promoter regions (Fig. 7).

## Discussion

A novel transcriptional regulator protein, BCARR, identified by purification, was found to have an affinity to the upstream regions of six proteolytic genes that were repressed in response to BCAAs in *L. helveticus* [10]. BCARR is thought to exert down-regulation in the proteolytic gene expressions by binding to the BCARR-box, 5′-AAAAANNCTWTTATT-3′ in the presence of BCAAs [10]. BCARR, first found in the proteolytic system of *L. helveticus*, seems to be a global regulator of amino acids metabolism because many gene expressions are broadly repressed by amino acids in CM4 [9]. However, the contribution of BCARR in all repressed gene expressions in the presence of amino acids remains unclear. Various approaches have been introduced in various bacterial gene expressions to study global regulatory genes. Specific DNA sequences for regulatory protein biding have been searched for genome-wide in *Escherichia coli* [15], *Sulfolobus acidophilus* [16], *Bacillus anthracis* [17], *Bacillus subtilis* [19, 21, 31–34], *Clostridium difficile* [35], *L. lactis* [18, 36, 37] and *Streptococcus thermophilus* [38]. Homologous sequences to the Cre-box sequence were searched for in the whole *Bacillus* genome and mapped on the genome, and the consensus sequence was newly deduced [33]. Currently, Cre-boxes are classified as high or low affinity sequences depending on the response at low and high levels of CcpAs characterized in *B. subtilis* [33].

A genome-wide analysis of BCARR-boxes was performed to understand the impact of BCARR on specific, 390 down-regulated genes by amino acids. For a more strategic analysis in the present study, BCARR-boxes located far from the promoters were considered to be less effective in repressing the gene expressions because BCARR can influence promoter activity by covering the surrounding promoter region ranging over approximately 200 bp of DNA [14]. Therefore, the BCARR-box was searched in the upstream regions from −300 to +250 bp at the promoter of the 390 down-regulated genes. The BCARR-box search at the promoter regions in the 390 repressed genes based on CLUSTALW analysis, 67 kinds of putative BCARR-boxes were found, especially at promoter regions of the proteolytic system, transporters and some transcriptional regulator genes among the 390 repressed genes (Table 1).

Among the predicted 67 genes with BCARR-boxes, 19 genes, shown on the right in Fig. 3 involved in the proteolytic system, amino acid and peptide transport system, transporters with a protease domain, and purine synthesis have a link to amino acid metabolism among the 33 annotated genes (57.5%) as illustrated in Fig. 3. These cell responses to excess amounts of intracellular amino acids seem to be a catabolite repression-like regulation because there is no need for more amino acid supply via these actions under nutrient-rich conditions. These results revealed that the BCARR system in *L. helveticus* might be the main regulatory system for the proteolytic system and transporters to link to amino acid metabolism as reported for the CodY system in *L. lactis* [12–14, 36, 37], *Bacillus subtilis* [11, 34] and *Streptococcus thermophilus* [38].

The right side of Fig. 3 shows that DapF, involved in L-lysine biosynthesis [39] from L-aspartate, may be controlled to decrease L-lysine production. GuaA and GuaB [31, 40] are involved in purine synthesis from IMP to GMP with conversion of Gln to Glu. So, BCARR may repress the supply of Glu throughout repressions of *guaA* and *guaB* gene expressions in amino acid rich conditions. For genes shown on the left side of Fig. 3, the reason for the repression of the gene expressions remains unclear. Repressions of some transporter genes, such as *deoR*, *lytL* and *lytR* [22, 41], are of interest, which will have a wide impact on many kinds of gene expressions. *DeoR* [22, 41] is present widely in Gram-positive and negative bacteria and acts as a repressor in sugar metabolism. The transcriptional regulators LytL and lytR were reported to be linked to *alaD* gene expression and may be involved in amino acid metabolism, but their role remains unclear. *cydC* [28, 42], *prtD* [29] and *uup* [43] gene products with ATPase activity have been suggested to contribute to membrane control against environmental stress. However, there is no clear evidence to explain a link between amino acid metabolism and these gene products.

To discern the repressive effects of the BCARR, all predicted motifs listed in Table 1 were mapped by location from the promoter and repressive effects (Fig. 5). The different repressive effects were thought to be caused by the location from the promoter, which influences RNA polymerase binding to the promoter, and/or by the preferred structural motif for BCARR. A BCARR-box was most frequently found in regulatory genes adjacent to the −35 sequence of the promoter regions of 67 proteolytic and some transporter genes (Fig. 4). Unexpectedly, the average repressive effects of the gene expressions through a BCARR-box with different locations were similar if the data were collected from a location ranging from −300 to 250 bp from the promoter (Fig. 4). A footprinting analysis in a previous study revealed a wide range of protection of the BCARR-box at the promoter region ranging over 200 bp of DNA by binding of the BCARR in the presence of amino acids [10]. Therefore, the binding of a BCARR to a BCARR-box located between −300 to +250 bp could be sufficient to interfere with the binding of the RNA polymerase to the promoter and repress the following transcription of the corresponding genes.

The structural features of the motif were more important than the location of the promoter because the distances of motifs from promoters did not influence repressive effects. To clarify the influence of the BCARR-box sequence on repressive effects, the structural features of BCARR-box sequences with high (Group A) and low (Group B) repressive effects were compared, and the number of base pairs in the expected palindromic structures was counted (Table 2). The average number of palindromic pairs was significantly higher in Group A (4.1 ± 0.8) than in Group B (3.3 ± 1.0) because of fewer Ts at positions 12 (67%), 13 (33%), 14 (67%) and 15 (67%) in Group B than in Group A. Moreover, the ΔG values were significantly lower in Group A (−0.25 ± 1.67) than in Group B (0.85 ± 0.90) (*P* < 0.05). These results suggest a more stable palindromic structure in the highly repressive Group A. This idea was also supported by the MEME analysis for all Group A and Group B sequences. The predicted motif for the less effective Group B, 5′-A1A2A3A4A5(W)6N7N8N9W10T11(T)12 W13(T)14(T)15–3′, showed more variable and fewer Ts at positions 12, 13, 14 and 15 (Fig. 6). These results suggest that a stable palindromic structure might have more frequent BCARR binding. For more precise analysis of the structure preferred by BCARR, a binding assay with purified BCARR toward each motif and a reporter assay involving each BCARR must be performed.

A genome-wide search revealed that higher gene repressions by amino acids might be distributed at some limited loci as shown by I to IV in Fig. 2 in the whole genome, whereas all predicted BCARR-box were distributed evenly in the whole genome in the present study (data not shown). The distributions of the potent repressions at the limited regions may be a more effective system in the acceleration of effective repressions of the neighboring genes. A wide range of unknown regulatory actions by amino acids may be involved in the gene repressions. A comparative analysis between the repressive effects measured like the whole cell response in the present study and a reporter assay containing the corresponding promotor regions may support this suggestion in *L. helveticus* CM4.

## Conclusion

The genome-wide search for the BCARR-box based on amino acid repressed genes in *L. helveticus* CM4 revealed frequent involvement in amino acid linked metabolism, such as the proteolytic system, transporter system, and some transcriptional regulator systems. The genes with more stable palindromic structures evaluated by BCARR-box motif analysis were preferable targets for BCARR binding and resulted in higher repressions in the target gene expressions. These results revealed that the BCARR system in *L. helveticus* might be the regulatory system of amino acid metabolism.

## Methods

### Strategic steps of genome-wide analysis

A transcriptome analysis was performed using CM4 cells collected 0.5 h after the addition of casein hydrolysate and the cells were compared to those without the peptides in the fermented milk. Genes down-regulated by peptides over 30% compared to control cells cultured without peptides were selected for the analysis of the BCARR-box in the corresponding genes (Step 1). Then, to select specific genes that were repressed by the binding of a BCARR to the BCARR-box, a homolog of the predicted BCARR-box was searched by CLUSTALW and LALIGN analyses in the repressed genes (Step 2). Next, the BCARR-box found in the promoter region of the repressed genes was analyzed for the relationship between the location at the promoter region and the repressive effect (Step 3). Then, the palindromic structures of the predicted BCARR-boxes in highly and lowly repressed groups were compared (Step 4).

### Bacterial strains and growth conditions

*L. helveticus* CM4 with strong extracellular proteinase activity [8] was pre-cultured in 100 ml of 9% (*W*/W) low-heated skimmed milk at 37 °C for 24 h. The pre-cultured fermented milk was added up to 5% of 1000 ml of fresh medium (9% low-heated skimmed milk). It was fermented at 37 °C for 25 h with the pH maintained at 6.0 by the addition of 50% NaOH, and the peptone (BD, NJ, USA) was added to the fermented milk up to 2% at 3 h after fermentation. For the transcriptome analysis of *L. helveticus* CM4 in fermented in milk medium with or without peptides, cells were harvested 0.5 h after the addition of peptone. Harvested cells were added into the same volume of RNA Protect Bacteria Reagent (Qiagen K. K., Tokyo, Japan).

### DNA microarray analysis

DNA microarray experiments were conducted using general protocols. Briefly, cells fermented in the skimmed milk medium with or without peptides were collected by centrifugation at 7000 rpm for 10 min. The cell pellets were quickly freeze in dry ice/ethanol and then stored at −30 °C for efficient cell lysis in the following step. Total RNA was extracted using an RNeasy Mini Kit (Qiagen, Valencia, CA) after lysis by lysozyme and phenol treatments. Total RNA was purified and used for a quality analysis. Untreated cells were used as controls. The microarray analysis was performed according to the protocol of Roche NimbleGen, Inc. (Madison, WI). A microarray for *L. helveticus* CM4 genes was prepared based on the whole genome sequence (unpublished sequence, but a related study was reported in ref. [20]) and was used in this study. cDNA was synthesized from total RNA for use in the hybridization. The hybridized arrays were scanned and normalized using NimbleScan software. The results for specific genes are presented as an n-fold change of expression. We selected genes if they were down-regulated less than 30% in a comparison of cells cultured with or without peptides.

## Additional file

Additional file 1: Table S1. Repressed gene expressions over 30% by adding peptides (amino acids) in CM4 (XLS 71 kb)

## Abbreviations

BCAAs: Branched chain amino acids
BCARR: Branched-chain amino acid responsive repressor
BCARR-box: Branched chain amino acid responsive repressor protein binding box
CodY-box: CodY protein binding box
